# Memory deficits in children and adolescents with a psychotic disorder: a systematic review and meta-analysis

**DOI:** 10.1007/s00406-025-01961-w

**Published:** 2025-02-04

**Authors:** Pilar de-la-Higuera-Gonzalez, Elisa Rodriguez-Toscano, Patricia Diaz-Carracedo, Maria Juliana Gonzalez-Urrea, Geraldine Padilla-Quiles, Marina Diaz-Marsa, Alejandro de la Torre-Luque

**Affiliations:** 1 Department of Personality, Assessment and Clinical Psychology, Universidad Complutense de Madrid (UCM), Madrid, Spain; 2 Health Research Institute, Hospital Clinico San Carlos (IdISSC), Universidad Complutense de Madrid (UCM), Madrid, Spain; 3 Faculty of Psychology, Department of Experimental Psychology, Cognitive Processes Language and Speech Therapy, Universidad Complutense de Madrid (UCM), Campus de Somosaguas. Ctra. de Húmera, S/N. Pozuelo de Alarcón, Madrid, Spain; 4 Department of Child and Adolescent Psychiatry, Hospital General Universitario Gregorio Marañon, Institute of Psychiatry and Mental Health (IiSGM), School of Medicine, Universidad Complutense (UCM), Madrid, Spain; 5 Universitat Oberta de Catalunya, Madrid, Spain; 6 Department of Legal Medicine, Psychiatry and Pathology, School of Medicine, Universidad Complutense de Madrid (UCM), Madrid, Spain; 7 Biomedical Research Networking Consortium for Mental Health (CIBERSAM ISCII), Madrid, Spain

**Keywords:** Early-onset psychosis, Verbal memory, Visual memory, Working memory, Short term memory, Long term memory

## Abstract

Early-onset psychosis (EOP) is a severe disorder which takes place before 18 years. It entails diverse clinical and functional implications, and it may lead to critical impairments in neurocognitive functions. Although deficits in memory are well described in adult populations and they appear to be clinically related with psychosis, impairments in memory in EOP show inconsistencies between studies. This study aimed to gain insight into the relationship between EOP and memory impairments, studying the potential contribution of moderators (storage source and memory content) on the observed memory deficits. This systematic review and meta-analysis was conducted following the PRISMA-2020 guidelines. Search was conducted in English and Spanish in five databases. Case–control studies which met all requirements were selected. Overall effect size was calculated under the random-effects model and Z-based tests were used. Heterogeneity was analysed by the *I*^2^ statistic. Mixed-effects meta-regression analysis was used to study the influence of methodological quality of studies, mean age, proportion of female participants within sample, mean diagnosis, memory storage type, memory content as moderators on individual effect size variability. As a result, 32 articles were finally selected, pooling data from 2636 participants (49.29% EOP participants). Overall effect size was Hedges’ *g* =  – 1.01, *CI*_95_ = [ – 1.35,  – 0.67], *p* < .01, indicating lower memory performance in the EOP group in comparison to healthy controls. Diagnosis and memory storage were found as significant moderators in the memory performance variance: larger deficits were found in children with psychosis and in working memory tasks.

**Supplementary Information:**

The online version contains supplementary material available at 10.1007/s00406-025-01961-w.

## Introduction

Schizophrenia spectrum and other psychotic disorders is a DSM-V category which comprises a wide variety of diagnostic entities covering psychotic syndromes. They are mainly featured by several primary symptoms, such as delusions, hallucinations, disorganised thought, disorganised motor behaviour and negative symptoms [1]. The onset of these disorders is usually observed in early adulthood or late adolescence [1]. It is named as early-onset psychosis (EOP) when the first psychotic episode takes place before 18 years old [2–4].

EOP has continuity with the adult psychotic form, in terms of clinical features and brain architecture [1, 5]. However, EOP may show greater clinical severity and a worse prognosis in clinical and functional terms with more severe symptoms and enduring impact in adulthood [1, 2, 4–7]. Moreover, EOP is associated with longer duration of untreated symptoms and premorbid social and occupational dysfunction including non-independent adult living, worse employment, low educational achievement, and social isolation [3, 7–9].

Cognitive symptoms in psychosis may represent a major unmet clinical need in patients with psychosis [10]. Neuropsychological deficits have been described before the onset of psychosis [11], in late prodromal states [12], in individuals at clinical high risk for psychosis [13] and persisting in every clinical state, regardless of the nature of the psychosis and illness progression [14, 15]. Deficits frequently observed among psychotic patients may compromise processing speed function, working memory, language, executive function, episodic memory, verbal memory, visual memory, processing speed, attention, inhibition and sensory processing [16]. Neurocognitive impairments have a strong relationship with social and occupational functioning [17] independently of age, gender, illness chronicity and inpatient status [18]. Regarding memory performance, a significant deficit has been observed in individuals with a first-episode psychosis (FEP), in terms of immediate verbal memory, delayed verbal and learning memory, nonverbal memory and working memory, being immediate episodic and verbal declarative memory largely impaired [14], even in drug-naïve patients [19]. Specifically, deficits in verbal memory tasks have been associated with higher risk of psychosis in individuals at clinical high risk [13] and therefore it may be considered a marker of disease progression in clinical high-risk individuals [12]. Also, difficulties in verbal memory have been associated with more severe positive symptoms in individuals in an early prodromal state [12]. Visuospatial working memory is significantly impaired in psychosis [20] even finding a more consistent and robust alteration than in verbal working memory [21]. Lower visual memory performance was associated with the presence of psychotic symptoms, independently of the general cognitive ability [22].

Specifically in EOP population, neurocognitive impairment seems to be a key clinical feature of EOP [23] and a broad range of cognitive deficits have been identified, with some inconsistencies between studies [4, 8, 24]. Despite mixed findings regarding relative severity and course, studies suggest that EOP may be associated with more severe cognitive deficits in comparison to adult-onset patients. This is present across various cognitive domains, such as slow processing speed, working memory, attention and executive functions [23, 25–28]. Moreover, longitudinal studies stress that the higher the neurocognitive deficits in EOP adolescents, the worse the prognosis of the disease throughout adulthood [5, 29]. Of particular interest, larger EOP impairments have been reported in both verbal memory and visual memory tasks [8, 30].

EOP patients may have significantly poorer performance than healthy controls (HC) in immediate verbal memory, short term verbal memory and long term verbal memory, existing deficits that may lead to a wider pattern of daily living dysfunction [31–33]. Moreover, a progressive impairment in verbal memory performance appeared to be associated with the development of severe and psychotic-level symptoms in EOP patients in comparison with other neurocognitive domains [34]. Significant impairments for visuospatial memory were also found in EOP patients [35, 36].

By and large, deficits in memory have been associated with worse functionality. EOP may be developed within an overall frame of vulnerability, in which neurocognitive functions are still under maturation, taking into account that adolescence is a sensitive neurodevelopment period [37–39]. In this regard, consolidation and refinement of top-down cognitive skills may be basically done throughout childhood and adolescence, in line with maturational processes of brain structures [28, 37, 40]. Besides, EOP may entail a deviation from the normal cognitive development [41], with substantial impact on key skills for real-world functioning, such as memory, abstract thinking and emotion regulation [3]. Although these deficits are widely described in previous meta-analyses [10, 14, 26], the impact of psychosis on memory storages and memory contents has not been specifically studied nor compared in EOP populations.

The present study aimed to gain insight into the memory deficits in EOP, because pharmacological and non-pharmacological cognitive-enhancing treatments appear to have inconsistent results in literature, in part because of the lack of consensus on assessment methods [42]. Thus, describing in detail cognitive deficits in EOP population is essential for fulfil that unmet in psychosis research. The identification of the more prevalent cognitive deficits in youth after a psychotic onset is key for the design of targeted therapeutic programmes. To achieve that goal, we investigated whether memory storages (working memory, short term vs. long term memory) may be distinctively affected by EOP (in comparison to HC) and to what extent this effect may vary in terms of memory content (i.e., verbal vs. visual content). Finally, it intended to explore other potential moderators in the relationship between memory deficits and the psychotic disorder in childhood and adolescents (e.g., sex, age, methodological quality of the studies). We hypothesised that the psychotic disorder -EOP- would show a far-reaching impact on performance in memory tasks, with a higher impact on long term memory and verbal content storage. On the other hand, the age of assessment would moderate this relationship.

## Methods

This study was conducted following the guidelines from the Preferred Reporting Items for Systematic Reviews and Meta-Analyses for Protocols (PRISMA-P, 2020) initiative [43]. Moreover, PRISMA checklist is included as supplementary material.

### Search strategy

Papers were located upon scientific database search. The consulted databases were: CINAHL, PsycInfo, PubMed, Redalyc, SCOPUS and Web of Science. Database search was conducted between December 2019 and June 2024. Queries were constructed using combinations of three main key terms and their respective thesaurus: psychosis and adolescent (or child) and memory. MeSH terms were also used in the PubMed database. See Table 1 to see the concrete search queries.

**Table 1:** Search queries

Database	Query	Hits
CINAHL	(schizophrenia OR psychotic OR psychosis) AND (child OR adolescent OR teenag*) AND (memory) [FILTERS: LANGUAGE English or Spanish]	148
PsycInfo	((schizophrenia OR psychotic OR psychosis) AND (child OR adolescent OR teenag*) AND (memory)) [FILTERS: LANGUAGE English or Spanish]	1674
PubMed	"Schizophrenia Spectrum and Other Psychotic Disorders"[Mesh] OR "Psychotic Disorders"[Mesh]) AND ("Memory"[Mesh] OR "Spatial Memory"[Mesh] OR "Memory, Episodic"[Mesh] OR "Memory, Long- Term"[Mesh] OR "Memory, Short-Term"[Mesh] OR "Memory Disorders"[Mesh] OR "Wechsler Memory Scale"[Mesh] OR "Memory and Learning Tests"[Mesh] OR "Memory Consolidation"[Mesh]) AND ("Child"[Mesh] OR "Adolescent"[Mesh]) "Schizophrenia Spectrum and Other Psychotic Disorders"[Mesh] OR "Psychotic Disorders"[Mesh]) AND "Amnesia, Anterograde"[Mesh] OR "Amnesia, Retrograde"[Mesh] OR "Amnesia"[Mesh]) AND ("Child"[Mesh] OR "Adolescent"[Mesh]) [FILTERS: LANGUAGE English or Spanish]	871
Redalyc	memoria AND niños OR adolescentes AND psicosis [FILTERS: LANGUAGE English or Spanish]	221
SCOPUS	(TITLE-ABS-KEY (schizophrenia OR psychotic OR psychosis) AND TITLE-ABS-KEY (child OR adolescent OR teenag*) AND TITLE-ABS-KEY ( memory)) AND (LIMIT-TO ( DOCTYPE, "ar")) AND ( LIMIT-TO (LANGUAGE, "English", “Spanish”) OR LIMIT-TO ( LANGUAGE, "Spanish")) AND ( EXCLUDE (EXACTKEYWORD, "Adult") OR EXCLUDE (EXACTKEYWORD, "Nonhuman") OR EXCLUDE ( EXACTKEYWORD, "Animals") OR EXCLUDE ( EXACTKEYWORD, "Animal Model"))	437
Web of Science	(TS = ((schizophrenia OR psychotic OR psychosis) AND (child OR adolescent OR teenag*) AND (memory or 'memory disorder')) NOT TS = ((ANIMAL OR ADULT))) AND LANGUAGE: (English OR Spanish) AND TYPE OF DOCUMENT: (Article) [FILTERS: LANGUAGE English or Spanish]	719

### Article selection criteria

Eligible articles were selected according to the *PICOS* strategy, which was used to define inclusion criteria for articles. Studies were selected if they met the criteria in five areas: Participants, Intervention, Control group, their Outcome and the type of Study. *Participants* of studies had with a diagnosis of schizophrenia or other psychotic disorders or bipolar disorder with psychotic symptoms according to the Diagnostic and Statistical Manual of Mental Disorders (DSM) criteria—DSM-IV-TR, or to the International Classification of Diseases (ICD) criteria—ICD-10 were considered. Also, studies were considered if the mean age of participants was below 18 years, or the onset of psychosis occurred at age 18 or younger. That is, the onset of the psychotic disorder was childhood or adolescence: either the upper limit of the range of age at onset was 18 years or the onset was defined by the authors as a ‘childhood onset’, ‘adolescence onset’, and/or ‘early-onset’. *Intervention* criteria involved studies in which participants were assessed for memory performance on a neuropsychological task, offering specific data of the performance in tasks for both clinical and control group. *Control* criteria guided the selection of studies which had a control group of healthy controls (HC). Regarding the *outcome* criteria, eligible studies were those which included a measure of memory performance through a task, validated or developed ad hoc. To consider the memory performance and taking into account the flexibility of the potential selected memory tasks, it was used Radvansky’s definition of memory, who considered it as ‘*the mental processes used to acquire (learn), store, or retrieve (remember) information*’ [44], [pp. 22–23]. So, the name of the memory task and the domain studied (type and memory storage) had to be included in the article for being the article considered in the current study. The type of *study* criteria referred to empirical case–control peer-reviewed original articles published in English or Spanish published in scientific literature from 2000 onwards.

The protocol for articles eligibility followed the next phases: Articles were firstly screened by reading title, abstract, and keywords. This phase was conducted by four independent researchers who reviewed each article to decide if it met the PICOS article selection criteria. This phase included the revision of 3129 articles. Articles which fulfilled inclusion criteria were pre-selected, and those which did not fitted with criteria were discarded. In the second phase, pre-selected papers were fully read to confirm the selection if they met the inclusion criteria. In case they did not, those articles were discarded. An independent reviewer (a senior researcher) endorsed the selection of each article that was ultimately included in the study. Any discrepancies in article selection were resolved through discussion, with the independent senior researcher, serving as the supervising author, acting as the final arbiter.

### Data extraction and bias assessment

Relevant data were extracted from each article using a coding manual, made purposely. Four different authors collected independently data from their reviewed reports following the coding manual. At the end of the process, one of the authors reviewed all the data collected, checking its accuracy with the original articles. Conflicts were resolved by discussion following the criteria of the supervisor authors. For the present study the following variables were considered relevant and were therefore included in the analysis: article’s year of publication, country of the corresponding author, sample size, mean age of participants, percentage of male/female participants within the study, sample size, type of psychotic disorder, comorbidity with physical and psychiatric disorders, type of memory assessed (working memory, short term and long term; see [45]), type of content (verbal vs. visual), instrument used to measure memory performance, and results of each assessed memory task obtained in the study.

The Newcastle – Ottawa Quality Assessment Scale (NOQAS; [46]) was used to measure methodological quality of studies to control for publication bias (besides funnel plots and other analytical strategies: see in the Data analysis section). The scale provides a quantitative measure of quality based on three domains: sample selection, exposure and comparability. A methodological quality score can be derived from the NOQAS ranging from 0 *(‘very weak level of methodological quality’*) to 9 *(‘very strong level of methodological quality’*). Four different authors assessed independently the methodological quality of studies to control for publication bias by the NOQAS. Conflicts were resolved by discussion following the criteria of the supervisor authors.

### Data analysis

First, individual effect size for each study (standardised mean difference estimate, comparing the memory performance between EOP and HC) were converted into the same metric (Hedges’ *g*). Second, we calculated the overall effect size under the random-effects model, taking into account a 95% confidence interval ([47], pp. 22–40). In studies with multiple effect size estimates, an overall effect size was calculated. Moreover, a z-based test was used to test whether the overall effect size was significantly different from zero (i.e., no effect). Forest plot was used to graphically display the individual effect sizes and the estimated overall one. Heterogeneity between individual effect sizes was analysed by using the Higgins and Thompson *I*^2^ statistic [48]. This statistic quantifies how severe is the heterogeneity (inconsistency) between individual effect size of studies, with *I*^2^ > 25% indicating low heterogeneity; *I*^2^ > 50% pointing to moderate heterogeneity; and *I*^2^ > 75% stating high heterogeneity between individual effect sizes.

The Egger’s regression asymmetry test and the contour-enhanced funnel plot were used to assess publication bias ([47], pp. 112–119). When the Egger’s regression *t* test supports the acceptance of the null hypothesis of symmetry in the funnel plot, the publication bias would not be upheld.

Mixed-effects meta-regression analysis was used to study the influence of quantitative moderators on individual effect size estimates. A model comparison was followed to study whether the solution with all the moderators in analysis (i.e., NOQAS score, mean age, proportion of female participants within sample, mean diagnosis, memory storage type, memory content) would fit better to data, than a model without moderators (i.e., unconstrained) and a model with sample and study feature moderators (i.e., NOQAS score, mean age, proportion of female participants within sample). The Akaike information criterion (AIC) was used for model comparison (i.e., the lower AIC, the better the fit to data). The random-effects maximum likelihood method was used for parameter estimation of moderator influence analysis.

All the analyses were conducted using the R × 64 3.0.1 software, with packages metafor and mvmeta.

## Results

**Fig. 1:**
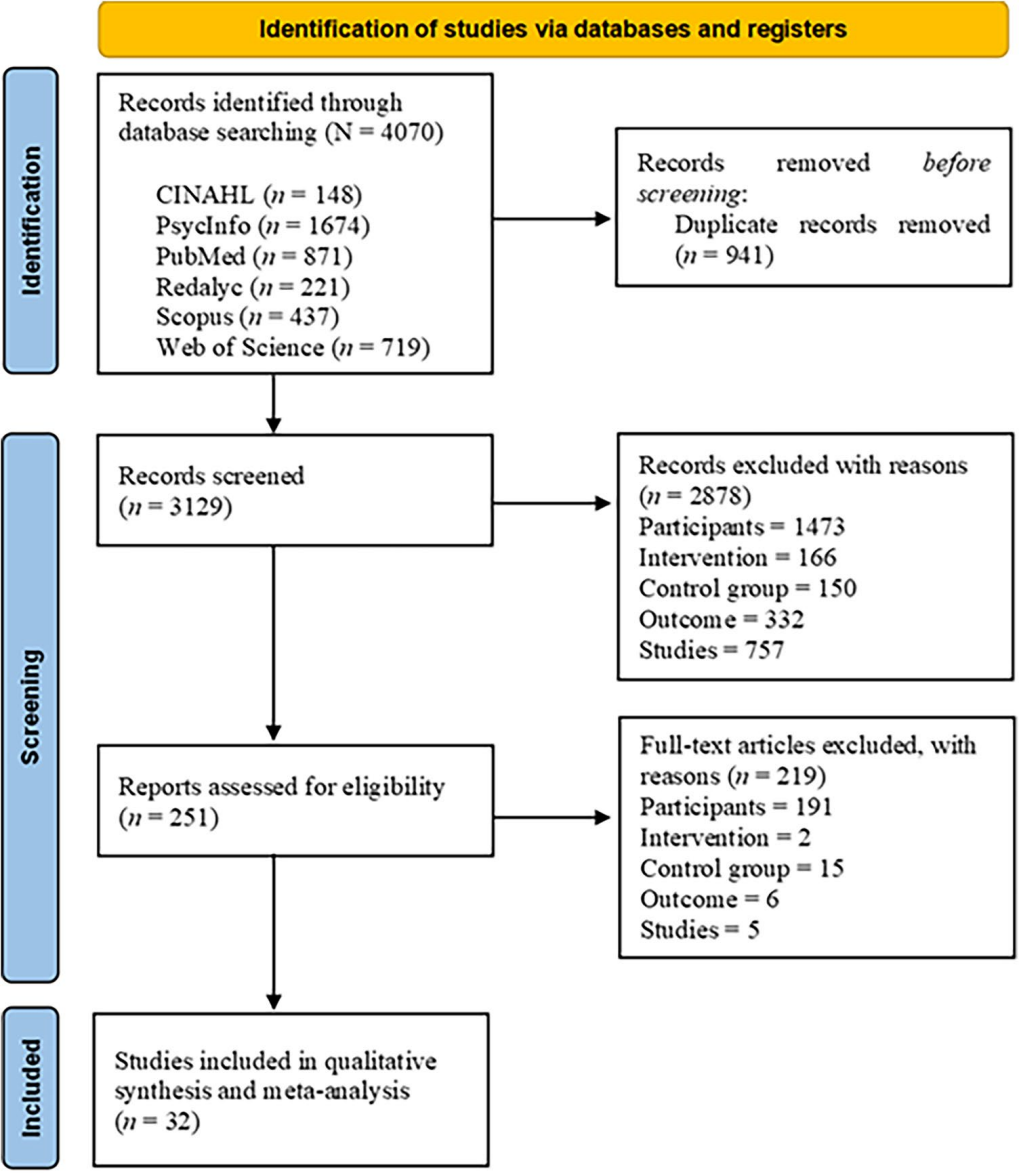
Flowchart of study selection

The Fig. 1 shows the flowchart of the study selection, displaying the process followed to select the articles to be reviewed. A sample of 32 articles was selected for this meta-analysis (see Table 2). Most studies (78.1% of studies) were conducted in the US, the United Kingdom, Norway, Spain and China (15.6% of studies each). 2010 was the year with the highest rate of published articles of this review (12.5% of articles) and most articles (71.8%) were published in 2010 onwards. Methodological quality of studies ranged from 5 to 9 stars (*M* = 7, *SD* = 1).

**Table 2:** Studies included in meta-analysis

Article	Country	Total sample	% Female sex	Mean age	Diagnosis	Memory test	Memory storage	Memory content	NOQAS score
Bachman et al. [49]	United States	66	31	16.63	Schizophrenia, schizoaffective and psychosis not otherwise specified	CVLT-II: Short & Long delay WMS-III: Spatial span, Visual Memory subtest (immediate & delayed recall)	STM & LTM	Verbal & visual	7
Bombin et al. [41]	Spain	154	33.77	15.43	Schizophrenia, bipolar disorder, psychosis not otherwise specified, schizoaffective disorder, major depression with psychotic symptoms, other affective disorders with psychotic symptoms, obsessive–compulsive disorder with psychotic symptoms	TAVEC: Short-Term Free Recall, Long-Term Free Recall, Discrimination WAIS-III: Digits Backward & Digits Forward, Number–Letter Sequencing	WM, STM & LTM	Verbal	6
Brodsky et al. [50]	United States	189	37.6	10.25	Children with childhood-onset Schizophrenia with or without ADHD comorbid disorder	SST	WM	Verbal	8
Chen et al. [51]	China	112	68.75	17.26	Schizophrenia, bipolar disorder, major depressive disorder	Working memory task^a^	WM	Visual	7
de la Serna et al. [52]	Spain	203	34.98	15.56	Schizophrenia, bipolar disorder, psychosis not otherwise specified, schizophreniform disorders, schizoaffective disorders, major depressions with psychotic features, brief reactive episodes, obsessive compulsive disorders with psychotic symptoms	TAVEC: Total learning, Short-term & Long-term free recall, Discrimination WAIS-III: Digits backward & Number-letter sequencing	WM, STM & LTM	Verbal	6
Dore et al. [53]	Canada	35	52.8	15.83	Schizophrenia, schizophreniform disorder, schizoaffective disorder and bipolar affective disorder with psychotic features	Memory task^b^	LTM	Verbal	8
Duan et al. [54]	China	96	35.38	15.4	Schizophrenia	HVLT-R	STM	Verbal	7
Frangou et al. [25]	United Kingdom	40	50	17.52	Schizophrenia	WMS-R: Delayed recall index	LTM	Verbal & visual	7
Guo et al. [55]	China	32	62.5	14.6	Bipolar disorder	DST: in forward & reverse order	WM & STM	Verbal & visual	7
Hintze & Borkowska [56]	Poland	63	41.5	17.2	Schizophrenia	N-back test RAVLT	WM & STM	Verbal & visual	7
Holmen et al. [57]	Norway	98	50	15,9	Schizophrenia, schizophreniform disorder, schizoaffective disorder, brief psychotic disorder, psychosis not otherwise specified	BVMT-R HVLT-R LNS WMS-III: Spatial Span	WM & STM	Verbal & visual	7
James et al. [58]	United Kingdom	61	42.62	16.1	Schizophrenia	RCFT: copy, immediate recall, delayed recall & recognition	STM & LTM	Verbal & visual	5
Jepsen et al. [59]	Denmark	131	71.75	15.89	Schizophrenia Non-affective psychosis	Brief Assessment of Cognition in Schizophrenia Cambridge Neuropsychological Test Automated Battery RCFT	WM, STM & LTM	Verbal & visual	6
Kravariti et al. [30]	United Kingdom	41	35	16.35	Schizophrenia	CEGT WMS-R: Verbal, Visual & General Memory, Delayed Recall	WM, STM & LTM	Verbal & visual	8
Kyriakopoulos et al. [60]	United Kingdom	45	42.22	16.2	Schizophrenia	Two back task	WM	Verbal	6
Landrø & Ueland [61]	Norway	49	38.1	15.25	Disorganized schizophrenia, paranoid schizophrenia, undifferentiated psychosis, schizoaffective disorder, schizotypal personality disorder	Multi-trial, free-recall paradigm ad hoc	STM & LTM	Verbal	8
Liang et al. [62]	China	83	45.78	14.28	Schizophrenia	BVMT-R HVLT-R	WM & STM	Verbal & visual	6
Mayoral et al. [27]	Spain	53	25	15.45	Schizophrenia, bipolar disorders, depression with psychotic features, schizoaffective disorder, schizophreniform disorder, psychosis not otherwise specified	TAVEC: Short Term Free Recall, Long Term Free Recall, Recognition, Total Learning; WAIS-III: Digits Backward, Number-Letter Sequencing	WM, STM & LTM	Verbal	9
Rhinewine et al. [63]	USA	106	37	15.8	Schizophrenia	CVLT: Total trials & delayed free recall WISC-III/WAIS-III: Digit span	STM	Verbal	7
Ruiz-Veguilla et al. [64]	Spain	119	45.4	16.5	Depressive disorder with psychotic symptoms, bipolar disorder, manic episode with psychotic symptoms, psychotic disorder not otherwise specified, schizophreniform disorder, schizophrenia, other psychotic disorders	RCF-II WISC-IV: Backward digit span test WMS-III: Immediate & delayed recall, Recognition	WM, STM & LTM	Verbal & visual	8
Sağlam et al. [65]	Turkey	150	54.72	17.4	Schizophrenia, Schizophreniform disorder, schizoaffective disorder, unspecified psychotic disorder, substance induced psychotic disorder	Auditory consonant trigrams RAVLT WMS-R	WM, STM, LTM	Verbal & visual	5
Thormodsen et al. [66]	Norway	30	53	16.05	Schizophrenia, schizophreniform disorder, schizoaffective disorder, brief psychotic disorder and psychosis not otherwise specified	N-back test	WM	Visual	8
Trotman et al. [67]	United States	89	46.07	14.33	Schizotypal personality disorder	WMS-III: Logical Memory immediate & delayed recall, Family Pictures immediate & delayed recall, Letter-Number Sequencing	WM	Verbal	8
Udal et al. [68]	Norway	23	56.52	13.3	Bipolar disorder	RAVLT: Short term & Long term recall, Recognition	STM & LTM	Verbal	5
Udal et al. [69]	Norway	24	58.33	13.9	Bipolar disorder	Knox Cube Test WISC-III / WAIS-III: Digit Span test	WM	Verbal & visual	8
Vance et al. [70]	Australia	47	0	14.05	Undifferenciated schizophrenia	CANTAB: Spatial Working Memory Between Search errors and Spatial Span tasks	WM	Visual	7
Vance et al. [35]	Australia	47	0	14.05	Undifferenciated schizophrenia	CANTAB: DMTS task (MTS trials & average delay trials)	WM & LTM	Visual	7
Walker & Standen [71]	United Kingdom	95	48.4	15.87	Schizophrenia, major affective disorder with comorbid psychoses	Camden Memory Test: Recognition of faces & words CnRep/AnRep CMS: Immediate, short-term & delayed recall Dot Task LNS Trail Making Test	WM, STM & LTM	Verbal	8
White et al. [72]	Netherlands	63	38.10	14.65	Schizophrenia, schizoaffective disorder, schizophreniform disorder	SIRP: Visuospatial & Verbal	WM	Verbal & visuospatial	7
White et al. [73]	USA	46	32.6	15	Schizophrenia, schizoaffective disorder, schizophreniform disorder	SIRP	WM	Verbal	8
Zabala et al. [28]	Spain	205	35.5	15.34	Schizophrenia, bipolar disorder, psychosis not otherwise specified, schizophreniform disorders, schizoaffective disorders, major depressions with psychotic features, brief reactive episodes, obsessive compulsive disorders with psychotic symptoms	TAVEC: Short term & Long term free recall, Discrimination, Total learning WAIS-III: Digits backwards, Number–letter sequencing	WM, STM & LTM	Verbal	7
Zhao et al. [74]	China	79	44.3	15.6	Schizophrenia	BVMT-R (total items recalled correctly after 3 trials) HVLT-R (total items recalled correctly after 3 trials)	STM	Verbal & visual	6

Note*.* *ADHD* Attention Deficit Hyperactivity Disorder, *AnRep* Adult Test of Nonword Repetition, *BVMT* Brief Visuospatial Memory Test, *BVMT-R* Brief Visuospatial Memory Test Revised, *CEGT* Computerised Executive Golf Task, *CMS* Children’s Memory Scale, *CnRep* The Children’s Test of Nonword Repetition, *CVLT-II* California Verbal Learning Test-Second Edition, *DST* Digit Span Test, *HVLT* Hopkins Verbal Learning Test, *HVLT-R* Hopkins Verbal Learning Test Revised, *LNS* Letter Number Span, *LTM* Long term memory, *NOQAS* Newcastle – Ottawa Quality Assessment Scale, *RAVLT* Rey’s Auditory Verbal Learning Test, *RCFT* Rey Complex Figure Test, *RCF-II* Spanish version of the Rey Complex Figure Test, *SIRP* Stenberg Item Recognition Paradigm, *SST* Sentence Span Task, *STM* Short term memory, *TAVEC* Spanish version of the California Verbal Learning Test, *WAIS-III* Wechsler Adult Intelligence Scale, 3rd Edition, *WAIS-IV* Wechsler Adult Intelligence Scale, 4th Edition, *WCST* Wisconsin Card Sorting, *WM* Working memory, *WISC-III* Wechsler Intelligence Scale for Children, 3rd Edition, *WMS-III* Wechsler Memory Scale-Third Edition, *WMS-R* Wechsler Memory Scale-Revised. ^a^In the working memory task, subjects were asked to respond as quickly as possible when they saw a number that appeared again only separated by one other number (i.e., 23–45– 23; participants responded to the second 23 as quickly as possible). ^b^The ad hoc memory task encompasses three encoding conditions: no cue (the word was read aloud by the experimenter), phonological cue /experimenter designated the word by its first syllable), and semantic cue (word was designed by its taxonomic category). Participants were instructed to learn the 15 words presented visually on a computer screen. The test phase consisted of a free recall and for the phonological and semantic cuing conditions a cued recall of the words not freely recalled was performed

Pooled sample of studies was 2636 participants (*M* = 82.38, *SD* = 52.06, range = 23–205), being 49.29% from EOP (*M* for the EOP = 40.56, *SD* = 29.75, range = 10–107; *M* for the HC = 37.47, *SD* = 23.59, range = 13–98). Mean proportion of female participants within samples was 42.55% (*SD* = 15.79, range = 0–71.75). Mean age was *M* = 15.4 years (*SD* = 1.42, range = 10.25–17.52). Regarding diagnoses, 90.7% of studies included adolescents with a schizophrenia spectrum and other psychotic disorders. Some of the subjects displayed comorbid disorders, such as attention deficit and hyperactivity disorder (0.77%). Regarding other comorbidities, in light of our results, 43.75% of the included articles did not mention comorbidity in relation to clinical participants, 40.63% of the articles used comorbidity as an exclusion criterion for the clinical sample and 15.62% of included articles considered comorbidity within the clinical participants. Among the articles that excluded participants with a concomitant Axis I disorder, 37.5% explicitly stated exclusion for substance use disorder, active substance use, or a history of substance use, and 6.12% of articles did not exclude clinical participants with substance use if a urine test was negative and psychotic symptoms remained active two weeks after the test. In the articles that considered comorbidity within the clinical participants, the comorbid disorders described were substance use disorders, anxiety, tic disorder, personality disorders, conduct disorder, developmental disorders, eating disorders and mood disorders. In terms of memory performance, 33.3% of studies included working memory tasks (Wechsler Adult Intelligence Scale, 3rd Edition -WAIS-III- subtests were the most common tools used), 35.7% of studies assessed immediate/short term memory and 31% of them delayed/long term memory (Rey’s Auditory Verbal Learning Test -RAVLT-subtests were the most common tools to assess short- and long term memory performance). Finally, 78.8% of effect sizes provided (*k* = 93) focused on verbal memory contents.

**Fig. 2:**
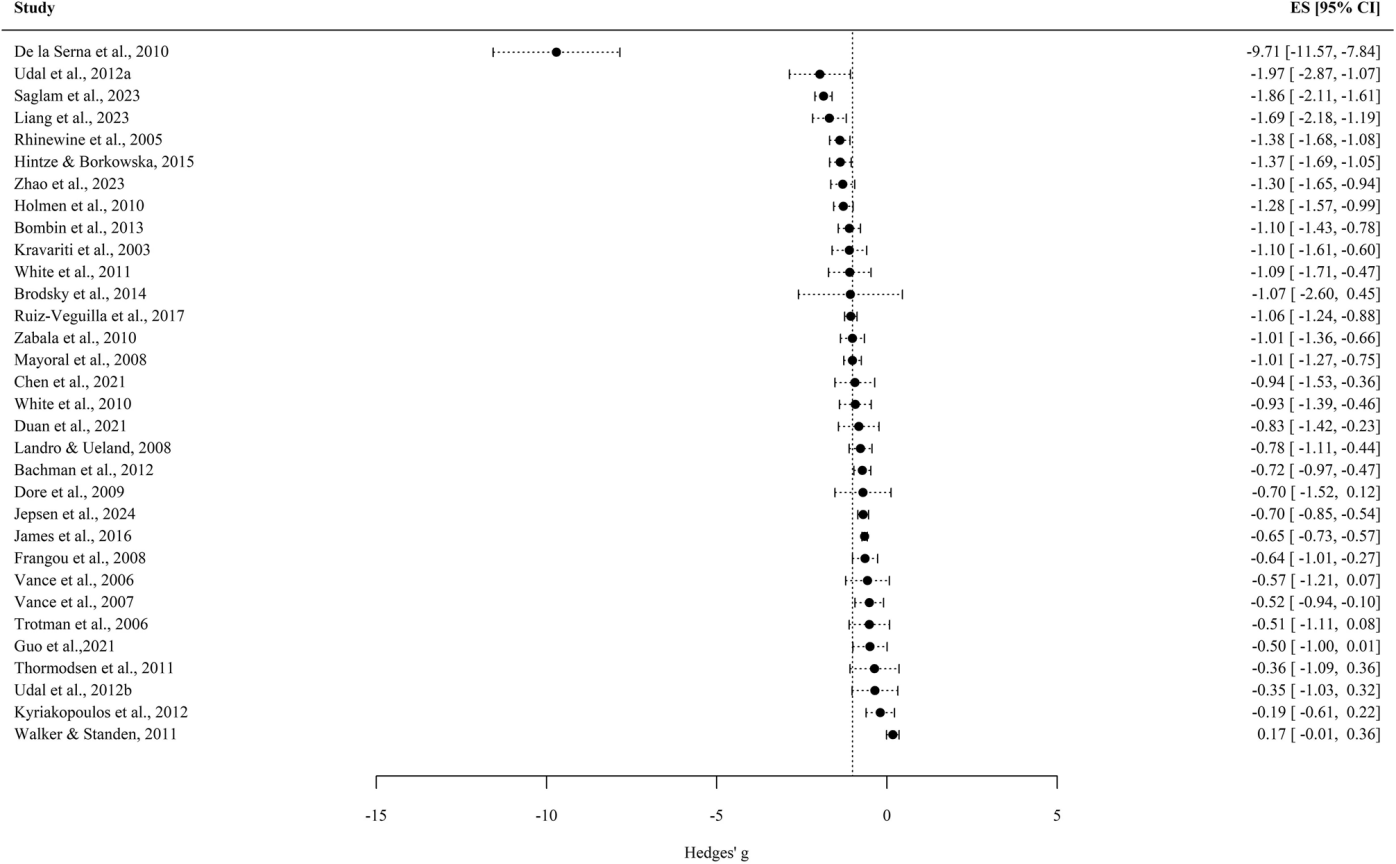
Forest plot of the individual effect sizes. Note. The vertical line represents the overall effect size (controlling for repeated estimates from a same study). Whisker bar represents the 95% confidence interval of individual estimates. *ES* Effect size, *CI* Confidence interval at 95%

The overall effect size derived from the studies and after controlling for repeated measures was *g* =  – 1.01, *CI*_95_ = [ – 1.35,  – 0.67], *Z* =  – 5.89, *p* < 0.01. This result points to lower levels of memory performance in the EOP (with a large effect size magnitude), in comparison to HC. The Fig. 2 displays the forest plot of the individual effect sizes: the pooled effect size (comprising the multiple estimates of this study) from the individual studies. Heterogeneity between the individual effect sizes was large, *Q* (120) = 33,905.52, *p* < 0.01; *I*^2^ = 99.6%. The study that showed the largest effect size was the one conducted by de la Serna et al. [52] (*g* =  – 9.71, *CI*_95_ = [ – 11.57,  – 7.84]) in which the influence of cannabis use was examined. By contrast, the study by Walker et al. [71] found the lack of a significant effect on memory performance, *g* = 0.17, *CI*_95_ = [ – 0.01, 0.36].

Regarding the publication bias for the overall estimate, the Egger’s test showed that the hypothesis of symmetry could not be upheld, *z* = – 15.36, *p* < 0.01. This result points that absence of publication bias could not be discarded on the results of this study; the results should be taken cautiously.

**Fig. 3:**
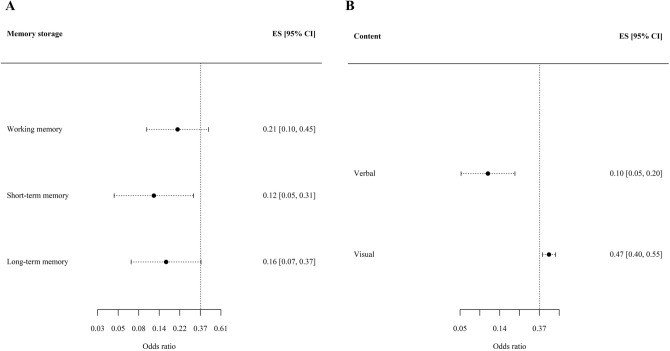
Forest plot of storage and memory-content effect sizes. Note. Box A represents the forest plot of memory storage moderators incorporated into this model (working memory, short-term memory and long-term memory). Box B represents the forest plot of memory content moderators incorporated into this model (verbal and visual). Category-specific effect size was calculated controlling for repeated measures from a same study, as well as the overall effect size (represented by the vertical line). Whisker bar represents the 95% confidence interval of individual estimates. *ES* Effect size, *CI* Confidence interval at 95%

In terms of moderators, the mixed-effect meta-regression analysis revealed that the model with all the moderators fitted better to data (AIC = 879.64), than the model without moderators (AIC = 8448.46) and the model with sociodemographic and methodological moderators (AIC = 906.3). Random-effects variance estimated in the model was σ^2^ = 1.67; and *Q*_*M*_ (7) = 38.66, *p* < 0.01. That means that the moderators incorporated into this model significantly explained the overall effect size variance. Two moderators showed a significant loading in the model: the disorder and the type of memory source. In terms of diagnosis, adolescents with a psychotic disorder showed a higher risk of memory deficits in comparison with those with a bipolar disorder with active or lifetime psychotic symptoms, *OR* = 0.35, *CI*_95_ = [0.13, 0.92], *z* =  – 2.12, *p* < 0.05. Moreover, deficits in immediate memory (*OR* = 0.71, *CI*_95_ = [0.63, 0.81], *z* =  – 5.12, *p* < 0.01) were milder than those observed in working memory tasks. Considering the three types of memory storage, the OR for short-term memory shows lower estimates than the overall effect size, including its confidence interval bounds. Moreover, poorer performance was observed in verbal memory tasks (*OR* = 0.10, *CI*_*95*_ = [0.05, 0.20]) compared to visual memory tasks (*OR* = 0.47, *CI*_*95*_ = [0.40, 0.55]). However, when relevant covariates were taken into account, no significant differences were observed based on content. This result points that individuals from the clinical group performed equally worse in both verbal memory tests and visual memory tests. The Fig. 3 displays the forest plot of storage (box A) and memory-content (box B) effect size: the effect size estimates by memory storage and memory content.

## Discussion

The present meta-analysis provides robust evidence on a deficit in memory in patients with EOP, coming from a pool of 32 studies and a sample of 2636 participants. Moreover, the study highlights higher risk of memory deficits in childhood and adolescents with schizophrenia spectrum and other psychotic disorders in comparison with those with a bipolar disorder with active or lifetime psychotic symptoms, equal impairments in verbal memory than in visual memory and results indicating the largest deficits on the working memory storage.

Strong deficits in memory performance of EOP patients were supported by the large overall effect size estimate (i.e., *g* =  – 1.01). In line with our results, lower levels of memory performance have been found in the studies assessing neuropsychological function among EOP patients [8, 25, 27, 41], even after symptom stabilisation [41] and without showing a progressive pattern [27].

Furthermore, the present study examined potential moderators that may show an influential role in the memory impairments of EOP patients, such as sex at birth, age, psychotic disorder diagnosis, memory storage, memory content and methodological quality of the reviewed studies. Meta-regression analysis showed that the aforementioned moderators substantially contributed to memory performance variance, as the model with covariates fitted better to data than an unconstrained model.

In terms of moderators, results stressed differential impairment across memory measures. The results showed milder deficits in immediate and delayed memory in comparison to working memory tasks. Deficits in working, short term and long term memory EOP have been described previously in case–control studies [27, 28, 41, 61]. When comparing memory storages, larger deficits in working memory have been found in EOP patients suggesting that working memory is the most affected memory storage after psychotic onset [8, 14, 26–28, 41, 61, 75]. Moreover, larger impairments in working memory have been found in people with schizophrenia and bipolar disorder with psychotic symptoms, in comparison with people with bipolar disorder without psychotic symptoms [76].

Cowan [45, 77, 78] posited that memory storage units show distinctive features, for instance in terms of temporal decay and chunk capacity limits. Accordingly, long term memory was conceptualised as a *‘vast store of knowledge and a record of prior events’* [45, pp. 324] and short term as the storage managing a *‘limited amount of information in a very accessible state temporarily’* [45, pp. 324]*.* Working memory -the component widely studied in reviewed studies- is understood as a component which mixes multiple storages at the same time, capable of working with information from long term memory, short term memory and other processing skills, temporarily sustaining information to make it more available into processing of the information which is taking place. Indeed, is it plausible that the lower memory performance observed in EOP patients and the significant differences between working and short term memory suggest that psychotic onset particularly affects the ability to integrate information from different storage systems and cognitive skills. Previous evidence has found that deficits in working memory and long term memory share a common neural substrate in schizophrenia suggesting that both deficits reflect the same functional disturbance. Altered function of the dorsolateral prefrontal cortex (DLPFC) has been proposed as an explanatory model for memory deficits in schizophrenia. Patients with schizophrenia exhibit abnormal activation of prefrontal cortex and temporal lobe structures during the performance of both working memory and long term memory tasks [79]. DLPFC is responsible for several functions associated with executive function, and its impairments can lead to deficits in tasks which require active representation of context information [16, 80]. Cognitive control can be differentiated in proactive control (goal information is actively maintained and interference is anticipated and prevented) and reactive control (detecting and resolving the interference once it has taken place) [81]. Working memory deficits may be considered as impairments in the active information maintenance and manipulation [80]. They have been related to deficits in the use of proactive control, which may lead to low activity encoding or maintaining information, impairing an effective response [16]. While the precise nature of these deficits remains unclear, the available data are most consistent with the view that patients activate an alternative network of brain regions than controls due to a yet unspecified deficit in the functional network deployed by healthy subjects during these tasks [82].

Taking into consideration the impact of psychosis on all mnesic storage components, it becomes essential to consider this global deficit due to the great impact of this decline in adolescents’ functioning. It could be related to a worse functioning in EOP population described in other studies [8, 29].

Regarding memory content, we found a similar deficit in memory tasks, regardless of whether they involved verbal or visual information. Participants with EOP exhibited significant deficits in both verbal and visual memory tests compared to control subjects. Our findings differ slightly from previous studies, which reported that verbal memory performance tends to be more strongly impaired [26, 61]. However, it is important to note that deficits in verbal learning have previously been linked to the age of onset [83]. The current study includes a homogeneous sample with a similar age of of the psychotic disorder onset, which may explain the comparable disruption of verbal and visual abilities. Furthermore, our results align with findings from a previous meta-analysis that investigated task differences in fMRI studies and found no significant effects on dorsolateral prefrontal cortex activation during memory tasks [82]. Another study that specifically examinated the working memory component identified posterior parietal hipoactivity, which could reflect a failure to manipulate and reorganize verbal and spatial information efficiently. This region’s involvement in working memory highlights its essential role in information manipulation [84].

Considering the diagnosis, adolescents with a psychotic disorder showed a higher risk of memory deficits in comparison with those with a bipolar disorder with active or lifetime psychotic symptoms. This result is consistent with those obtained by other meta-analyses in non-adult population such as from [26]. They found larger deficits in memory performance in children with schizophrenia spectrum than in children with bipolar disorder (for working memory: psychosis group *ES* =  – 0.99, bipolar group *ES* =  – 0.68; for verbal learning and memory: psychosis group *ES* =  – 0.86, bipolar group *ES* =  – 0.83; for visual memory: psychosis group *ES* =  – 0.82, bipolar group ES =  – 0.44), and they differ in a statistically significative way for Working Memory and for Visual Working Memory. Similar results have been found on adult patients [85, 86], finding worse results in memory tasks -Working memory, Visual and Verbal Learning- by Schizophrenia patients than those by Bipolar Patients [86]. It agrees with the dimensional vision of psychotic disorders, in which people with schizophrenia show larger deficits than bipolar patients.

On the other hand, the sample’s age did not significantly contribute to the overall effect size variance, either. A previous meta-analysis [10] found that the severity of cognitive deficits varies according to the age of onset of the psychotic disorder, highlighting that an earlier age of onset is associated with stronger deficits in memory [75, 87]. Our results are not aligned with those from the Rajji’s meta-analysis, mainly because of the targeted population (adolescents with EOP). In this regard, great severity of psychosis with an adolescent onset is described through studies [2, 4, 5]. It may involve that memory deficits may constitute a key feature of EOP, regardless of clinical profile. In relation to sex effect, similarly to previous studies with EOP population [64], our results did not find a specific effect of sex in memory storages nor regarding memory content. Similarly, the methodological quality of the studies did not show a significant effect in explaining memory performance variance. This may be related with the low variability between studies in terms of methodological quality, as most of them showed key controls against internal validity threads.

Results from our study have been visualised by following a robust procedure, under the compliance of international guidelines for systematic review and meta-analysis. A wide number of databases were searched. Moreover, a mixed-effects approach was followed (it allows for setting key analytical controls in terms of within- and between-study effects). However, this meta-analysis also has some limitations to be mentioned. First, results from this meta-analysis should be taken cautiously, as they could be influenced by publication bias effects. Although there were employed the NOQAS and the Egger’s regression asymmetry test and the contour-enhanced funnel plot, further bias risk assessment could had been included. Second, we did not consider substance use as a moderator for memory performance in EOP patients. In this regard, [88] found a strong relationship between cannabis use and memory deficits of young people with schizophrenia. In the context of our meta-analysis (i.e., memory performance in EOP adolescents), only one primary study provided data on cannabis use [52], with the subsequent impossibility to conduct any sensitivity analysis on this moderator. Further empirical research should be done on the relationship between substance use and memory performance in this particular population. Likewise, pharmacological treatment has not been taken into account as a moderator. Although pharmacological treatment is known to impact cognition [89], EOP patients commonly have a little cumulative dose of antipsychotic medication or are even naive to medication. Two of the studies reviewed in the present meta-analysis provided some supplementary analysis on antipsychotic medication in EOP patients, finding a null effect on memory performance [27, 28]. Additionally, it should be considered that grey literature studies have not been reviewed, due to its expected lower methodological quality, with the related difficulty to attribute memory performance effects from moderators of interest. Finally, regarding the literature search, it must be borne in mind that the current search is limited to articles in English and Spanish since 2000. Although English and Spanish are two predominant languages in scientific literature, and 2000 is a suitable starting point to explore memory in EOS through more recent research, this restriction may limit the number of articles screened and considered.

In terms of clinical implications, this study provides some robust data on the universal deficit in memory skills of EOP patients. Although an effort in developing pharmacological and non-pharmacological cognitive-enhancing treatments for psychotic patients, results and efficacy of treatments are inconsistently reported in literature. Distinguishing the impact of the EOP cognitive deficits on the three memory storages and considering the memory content, may allow determining the specific deficits more prevalent in young patients after the psychotic onset. It could improve therapeutic programmes by targeting the main important deficits of the disorder, such as verbal memory levels of performance, that may decisively contribute to improve patient’s psychosocial functionality. Cognitive remediation may become an effective approach with satisfactory results in terms of cognition and functioning in EOP, observing substantial improvements in working memory [90]. In light of the current results, therapeutic programmes should be developed considering the global deficit in memory.

To sum up, this meta-analysis compiles current evidence into psychosis’s effect on memory, highlighting that EOP may strongly affect memory skills, specially working memory storage. This knowledge is essential to be considered, due to the opportunity to consider mnesic deficits as target points in therapeutic programmes, which allow children and adolescents to improve their memory capacities and to raise their abilities, achieving a better functioning and adaptation.

## Supplementary Information

Below is the link to the electronic supplementary material.

Supplementary file1 (DOCX 36 KB)

## Data Availability

The data will be made available upon reasonable request to the authors.
