# Correction: Memory deficits in children and adolescents with a psychotic disorder: a systematic review and meta-analysis

**DOI:** 10.1007/s00406-025-02137-2

**Published:** 2025-11-11

**Authors:** Pilar de-la-Higuera-Gonzalez, Elisa Rodriguez-Toscano, Patricia Diaz-Carracedo, Maria Juliana Gonzalez-Urrea, Geraldine Padilla-Quiles, Marina Diaz-Marsa, Alejandro de la Torre-Luque

**Affiliations:** 1 Department of Personality, Assessment and Clinical Psychology, Universidad Complutense de Madrid (UCM), Madrid, Spain; 2 Health Research Institute, Hospital Clinico San Carlos (IdISSC), Universidad Complutense de Madrid (UCM), Madrid, Spain; 3 Faculty of Psychology, Department of Experimental Psychology, Cognitive Processes Language and Speech Therapy, Universidad Complutense de Madrid (UCM), Campus de Somosaguas. Ctra. de Húmera, S/N. Pozuelo de Alarcón, Madrid, Spain; 4 Department of Child and Adolescent Psychiatry, Hospital General Universitario Gregorio Marañon, Institute of Psychiatry and Mental Health (IiSGM), School of Medicine, Universidad Complutense (UCM), Madrid, Spain; 5 Universitat Oberta de Catalunya, Madrid, Spain; 6 Department of Legal Medicine, Psychiatry and Pathology, School of Medicine, Universidad Complutense de Madrid (UCM), Madrid, Spain; 7 Biomedical Research Networking Consortium for Mental Health (CIBERSAM ISCII), Madrid, Spain

**Correction: European Archives of Psychiatry and Clinical Neuroscience (2025) 275:715–732**

10.1007/s00406-025-01961-w

The article “Memory deficits in children and adolescents with a psychotic disorder: a systematic review and meta-analysis”, written by Pilar de‑la‑Higuera‑Gonzalez, Elisa Rodriguez‑Toscano, Patricia Diaz-Carracedo, Maria Juliana Gonzalez-Urrea, Geraldine Padilla-Quiles, Marina Diaz Marsa, Alejandro de la Torre-Luque was originally published under exclusive license to © Springer-Verlag GmbH Germany, part of Springer Nature 2025. As a result of the subsequent decision to publish the article under the open access model, the article’s copyright notice was changed on October, 9th, 2025 to © The Author(s) 2025 and the article is now distributed under a Creative Commons Attribution-NonCommercial-NoDerivatives 4.0 International License, which permits any non-commercial use, sharing, distribution and reproduction in any medium or format, as long as you give appropriate credit to the original author(s) and the source, provide a link to the Creative Commons licence, and indicate if you modified the licensed material. You do not have permission under this licence to share adapted material derived from this article or parts of it. The images or other third party materials in this article are included in the article’s Creative Commons licence, unless indicated otherwise in a credit line to the material. If material is not included in the article’s Creative Commons licence and your intended use is not permitted by statutory regulation or exceeds the permitted use, you will need to obtain permission directly from the copyright holder. To view a copy of this licence, visit http://creativecommons.org/licenses/by-nc-nd/4.0/.

